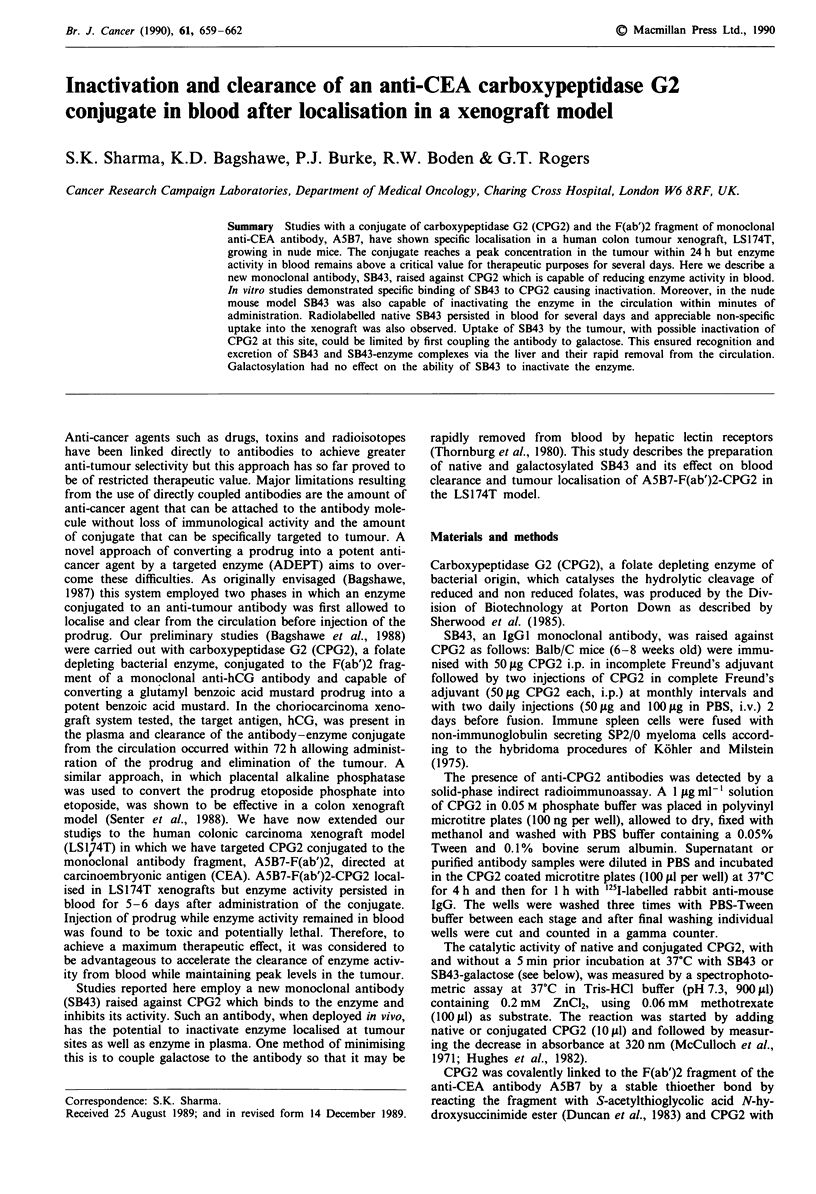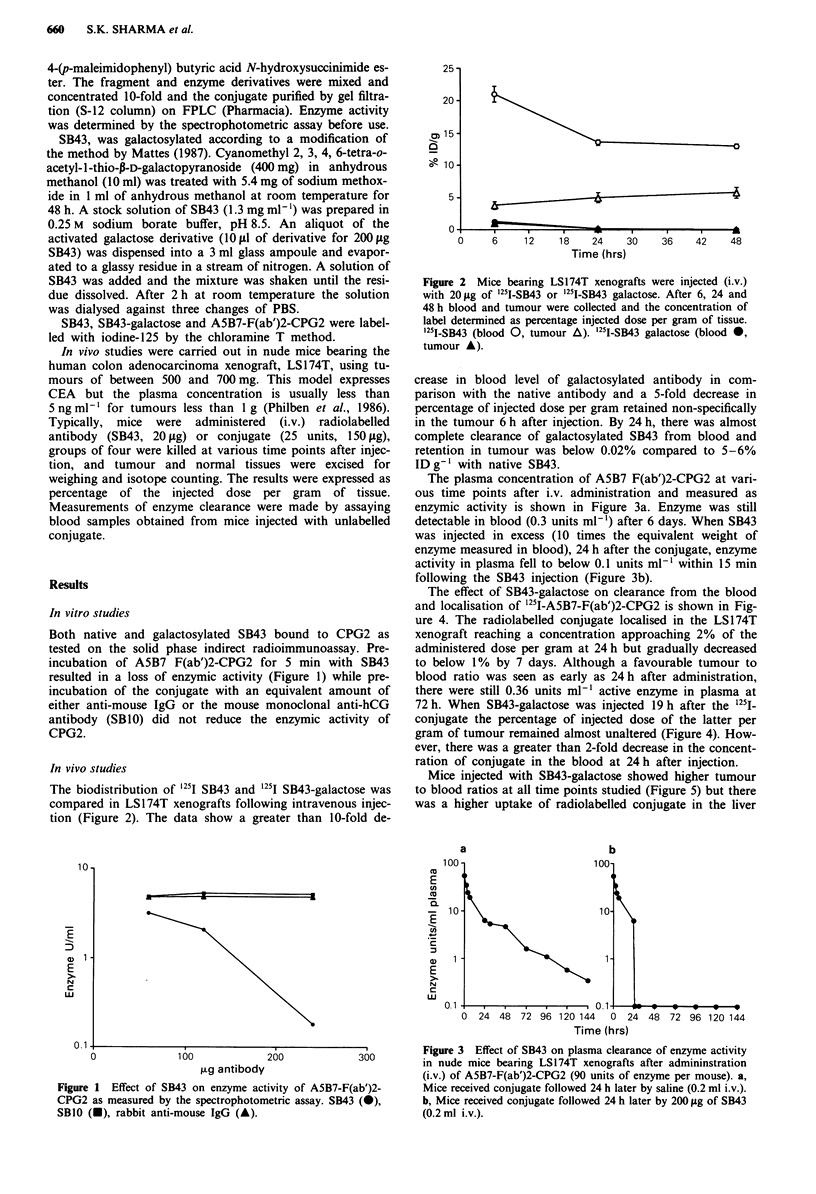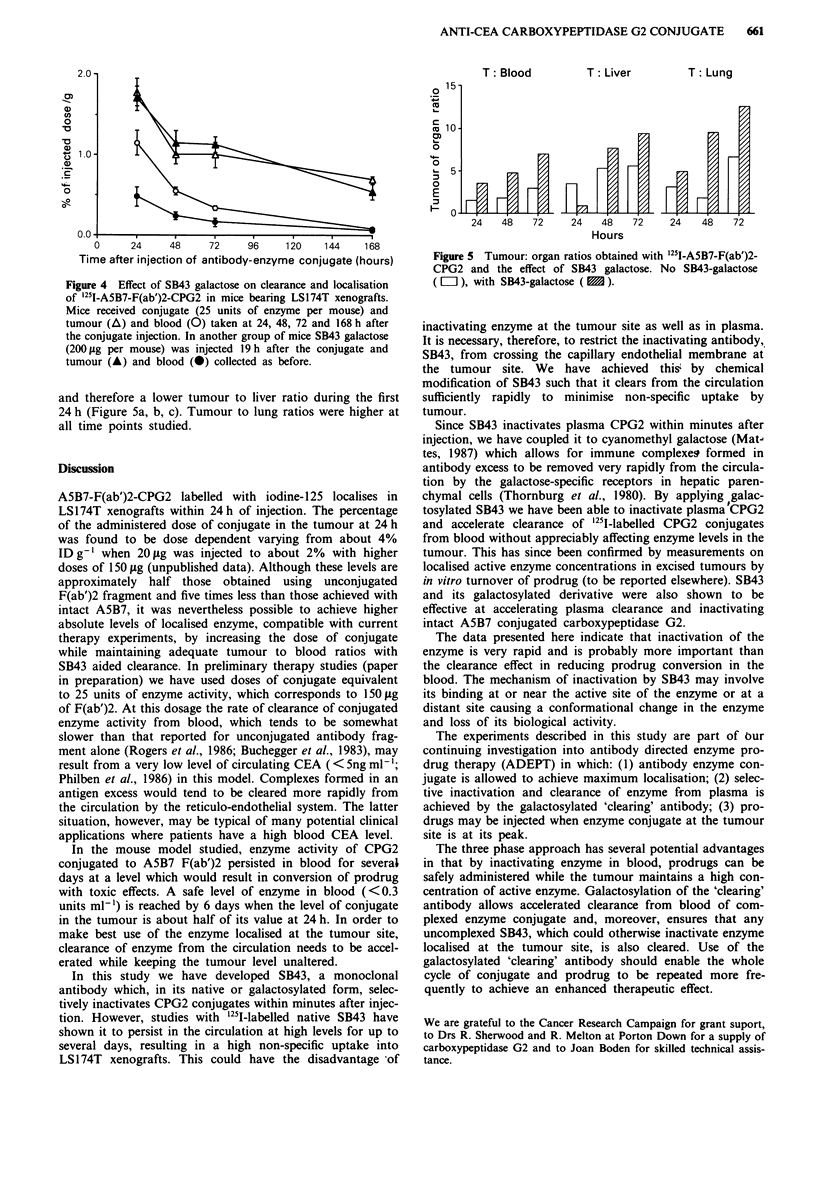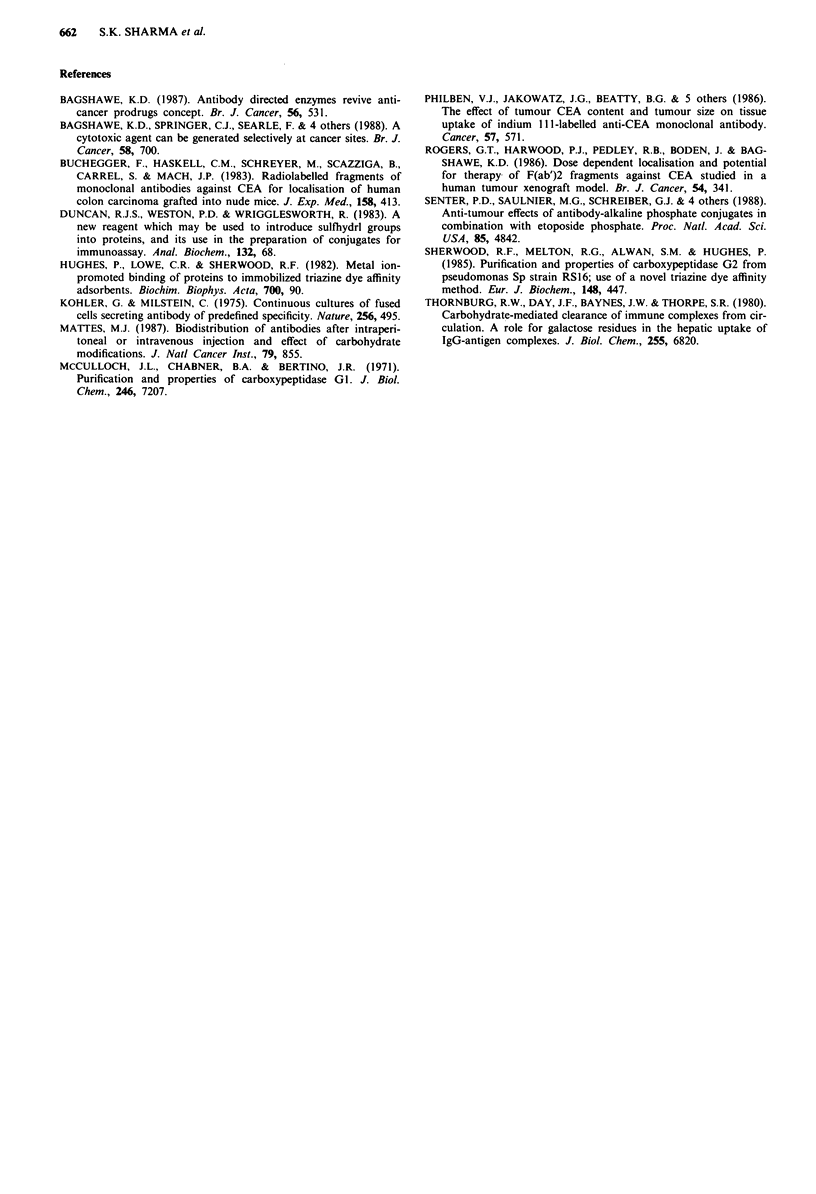# Inactivation and clearance of an anti-CEA carboxypeptidase G2 conjugate in blood after localisation in a xenograft model.

**DOI:** 10.1038/bjc.1990.149

**Published:** 1990-05

**Authors:** S. K. Sharma, K. D. Bagshawe, P. J. Burke, R. W. Boden, G. T. Rogers

**Affiliations:** Department of Medical Oncology, Charing Cross Hospital, London, UK.

## Abstract

Studies with a conjugate of carboxypeptidase G2 (CPG2) and the F(ab')2 fragment of monoclonal anti-CEA antibody, A5B7, have shown specific localisation in a human colon tumour xenograft, LS174T, growing in nude mice. The conjugate reaches a peak concentration in the tumour within 24 h but enzyme activity in blood remains above a critical value for therapeutic purposes for several days. Here we describe a new monoclonal antibody, SB43, raised against CPG2 which is capable of reducing enzyme activity in blood. In vitro studies demonstrated specific binding of SB43 to CPG2 causing inactivation. Moreover, in the nude mouse model SB43 was also capable of inactivating the enzyme in the circulation within minutes of administration. Radiolabelled native SB43 persisted in blood for several days and appreciable non-specific uptake into the xenograft was also observed. Uptake of SB43 by the tumour, with possible inactivation of CPG2 at this site, could be limited by first coupling the antibody to galactose. This ensured recognition and excretion of SB43 and SB43-enzyme complexes via the liver and their rapid removal from the circulation. Galactosylation had no effect on the ability of SB43 to inactivate the enzyme.


Br. J. Cancer (1990), 61, 659–662 © Macmillan Press Ltd., 1990

### Inactivation and clearance of an anti-CEA carboxypeptidase G2 conjugate in blood after localisation in a xenograft model

S.K. Sharma, K.D. Bagshawe, P.J. Burke, R.W. Boden & G.T. Rogers

*Cancer Research Campaign Laboratories, Department of Medical Oncology, Charing Cross Hospital, London W6 8RF, UK.*

**Summary** Studies with a conjugate of carboxypeptidase G2 (CPG2) and the F(ab')2 fragment of monoclonal anti-CEA antibody, A5B7, have shown specific localisation in a human colon tumour xenograft, LS174T, growing in nude mice. The conjugate reaches a peak concentration in the tumour within 24 h but enzyme activity in blood remains above a critical value for therapeutic purposes for several days. Here we describe a new monoclonal antibody, SB43, raised against CPG2 which is capable of reducing enzyme activity in blood. *In vitro* studies demonstrated specific binding of SB43 to CPG2 causing inactivation. Moreover, in the nude mouse model SB43 was also capable of inactivating the enzyme in the circulation within minutes of administration. Radiolabelled native SB43 persisted in blood for several days and appreciable non-specific uptake into the xenograft was also observed. Uptake of SB43 by the tumour, with possible inactivation of CPG2 at this site, could be limited by first coupling the antibody to galactose. This ensured recognition and excretion of SB43 and SB43-enzyme complexes via the liver and their rapid removal from the circulation. Galactosylation had no effect on the ability of SB43 to inactivate the enzyme.

Anti-cancer agents such as drugs, toxins and radioisotopes have been linked directly to antibodies to achieve greater anti-tumour selectivity but this approach has so far proved to be of restricted therapeutic value. Major limitations resulting from the use of directly coupled antibodies are the amount of anti-cancer agent that can be attached to the antibody molecule without loss of immunological activity and the amount of conjugate that can be specifically targeted to tumour. A novel approach of converting a prodrug into a potent anti-cancer agent by a targeted enzyme (ADEPT) aims to overcome these difficulties. As originally envisaged (Bagshawe, 1987) this system employed two phases in which an enzyme conjugated to an anti-tumour antibody was first allowed to localise and clear from the circulation before injection of the prodrug. Our preliminary studies (Bagshawe *et al.*, 1988) were carried out with carboxypeptidase G2 (CPG2), a folate depleting bacterial enzyme, conjugated to the F(ab')2 fragment of a monoclonal anti-hCG antibody and capable of converting a glutamyl benzoic acid mustard prodrug into a potent benzoic acid mustard. In the choriocarcinoma xenograft system tested, the target antigen, hCG, was present in the plasma and clearance of the antibody–enzyme conjugate from the circulation occurred within 72 h allowing administration of the prodrug and elimination of the tumour. A similar approach, in which placental alkaline phosphatase was used to convert the prodrug etoposide phosphate into etoposide, was shown to be effective in a colon xenograft model (Senter *et al.*, 1988). We have now extended our studies to the human colonic carcinoma xenograft model (LS174T) in which we have targeted CPG2 conjugated to the monoclonal antibody fragment, A5B7-F(ab')2, directed at carcinoembryonic antigen (CEA). A5B7-F(ab')2-CPG2 localised in LS174T xenografts but enzyme activity persisted in blood for 5–6 days after administration of the conjugate. Injection of prodrug while enzyme activity remained in blood was found to be toxic and potentially lethal. Therefore, to achieve a maximum therapeutic effect, it was considered to be advantageous to accelerate the clearance of enzyme activity from blood while maintaining peak levels in the tumour.

Studies reported here employ a new monoclonal antibody (SB43) raised against CPG2 which binds to the enzyme and inhibits its activity. Such an antibody, when deployed *in vivo*, has the potential to inactivate enzyme localised at tumour sites as well as enzyme in plasma. One method of minimising this is to couple galactose to the antibody so that it may be rapidly removed from blood by hepatic lectin receptors (Thornburg *et al.*, 1980). This study describes the preparation of native and galactosylated SB43 and its effect on blood clearance and tumour localisation of A5B7-F(ab')2-CPG2 in the LS174T model.

Correspondence: S.K. Sharma.
Received 25 August 1989; and in revised form 14 December 1989.

### Materials and methods

Carboxypeptidase G2 (CPG2), a folate depleting enzyme of bacterial origin, which catalyses the hydrolytic cleavage of reduced and non reduced folates, was produced by the Division of Biotechnology at Porton Down as described by Sherwood *et al.* (1985).

SB43, an IgG1 monoclonal antibody, was raised against CPG2 as follows: Balb/C mice (6–8 weeks old) were immunised with 50 μg CPG2 i.p. in incomplete Freund's adjuvant followed by two injections of CPG2 in complete Freund's adjuvant (50 μg CPG2 each, i.p.) at monthly intervals and with two daily injections (50 μg and 100 μg in PBS, i.v.) 2 days before fusion. Immune spleen cells were fused with non-immunoglobulin secreting SP2/0 myeloma cells according to the hybridoma procedures of Köhler and Milstein (1975).

The presence of anti-CPG2 antibodies was detected by a solid-phase indirect radioimmunoassay. A 1 $\mu g\ ml^{-1}$ solution of CPG2 in 0.05 M phosphate buffer was placed in polyvinyl microtitre plates (100 ng per well), allowed to dry, fixed with methanol and washed with PBS buffer containing a 0.05% Tween and 0.1% bovine serum albumin. Supernatant or purified antibody samples were diluted in PBS and incubated in the CPG2 coated microtitre plates (100 μl per well) at 37°C for 4 h and then for 1 h with $^{125}I$-labelled rabbit anti-mouse IgG. The wells were washed three times with PBS-Tween buffer between each stage and after final washing individual wells were cut and counted in a gamma counter.

The catalytic activity of native and conjugated CPG2, with and without a 5 min prior incubation at 37°C with SB43 or SB43-galactose (see below), was measured by a spectrophotometric assay at 37°C in Tris-HCl buffer (pH 7.3, 900 μl) containing 0.2 mM $ZnCl_2$, using 0.06 mM methotrexate (100 μl) as substrate. The reaction was started by adding native or conjugated CPG2 (10 μl) and followed by measuring the decrease in absorbance at 320 nm (McCulloch *et al.*, 1971; Hughes *et al.*, 1982).

CPG2 was covalently linked to the F(ab')2 fragment of the anti-CEA antibody A5B7 by a stable thioether bond by reacting the fragment with *S*-acetylthioglycolic acid *N*-hydroxysuccinimide ester (Duncan *et al.*, 1983) and CPG2 with

4-(*p*-maleimidophenyl) butyric acid *N*-hydroxysuccinimide ester. The fragment and enzyme derivatives were mixed and concentrated 10-fold and the conjugate purified by gel filtration (S-12 column) on FPLC (Pharmacia). Enzyme activity was determined by the spectrophotometric assay before use.

SB43, was galactosylated according to a modification of the method by Mattes (1987). Cyanomethyl 2, 3, 4, 6-tetra-*o*-acetyl-1-thio-β-D-galactopyranoside (400 mg) in anhydrous methanol (10 ml) was treated with 5.4 mg of sodium methoxide in 1 ml of anhydrous methanol at room temperature for 48 h. A stock solution of SB43 (1.3 mg $ml^{-1}$) was prepared in 0.25 M sodium borate buffer, pH 8.5. An aliquot of the activated galactose derivative (10 µl of derivative for 200 µg SB43) was dispensed into a 3 ml glass ampoule and evaporated to a glassy residue in a stream of nitrogen. A solution of SB43 was added and the mixture was shaken until the residue dissolved. After 2 h at room temperature the solution was dialysed against three changes of PBS.

SB43, SB43-galactose and A5B7-F(ab')2-CPG2 were labelled with iodine-125 by the chloramine T method.

*In vivo* studies were carried out in nude mice bearing the human colon adenocarcinoma xenograft, LS174T, using tumours of between 500 and 700 mg. This model expresses CEA but the plasma concentration is usually less than 5 ng $ml^{-1}$ for tumours less than 1 g (Philben *et al.*, 1986). Typically, mice were administered (i.v.) radiolabelled antibody (SB43, 20 µg) or conjugate (25 units, 150 µg), groups of four were killed at various time points after injection, and tumour and normal tissues were excised for weighing and isotope counting. The results were expressed as percentage of the injected dose per gram of tissue. Measurements of enzyme clearance were made by assaying blood samples obtained from mice injected with unlabelled conjugate.

### Results

### *In vitro studies*

Both native and galactosylated SB43 bound to CPG2 as tested on the solid phase indirect radioimmunoassay. Preincubation of A5B7 F(ab')2-CPG2 for 5 min with SB43 resulted in a loss of enzymic activity (Figure 1) while preincubation of the conjugate with an equivalent amount of either anti-mouse IgG or the mouse monoclonal anti-hCG antibody (SB10) did not reduce the enzymic activity of CPG2.

### *In vivo studies*

The biodistribution of $^{125}$I SB43 and $^{125}$I SB43-galactose was compared in LS174T xenografts following intravenous injection (Figure 2). The data show a greater than 10-fold decrease in blood level of galactosylated antibody in comparison with the native antibody and a 5-fold decrease in percentage of injected dose per gram retained non-specifically in the tumour 6 h after injection. By 24 h, there was almost complete clearance of galactosylated SB43 from blood and retention in tumour was below 0.02% compared to 5–6% ID $g^{-1}$ with native SB43.

**Figure 2** Mice bearing LS174T xenografts were injected (i.v.) with 20 µg of $^{125}$I-SB43 or $^{125}$I-SB43 galactose. After 6, 24 and 48 h blood and tumour were collected and the concentration of label determined as percentage injected dose per gram of tissue. $^{125}$I-SB43 (blood ○, tumour △). $^{125}$I-SB43 galactose (blood ●, tumour ▲).

The plasma concentration of A5B7 F(ab')2-CPG2 at various time points after i.v. administration and measured as enzymic activity is shown in Figure 3a. Enzyme was still detectable in blood (0.3 units $ml^{-1}$) after 6 days. When SB43 was injected in excess (10 times the equivalent weight of enzyme measured in blood), 24 h after the conjugate, enzyme activity in plasma fell to below 0.1 units $ml^{-1}$ within 15 min following the SB43 injection (Figure 3b).

The effect of SB43-galactose on clearance from the blood and localisation of $^{125}$I-A5B7-F(ab')2-CPG2 is shown in Figure 4. The radiolabelled conjugate localised in the LS174T xenograft reaching a concentration approaching 2% of the administered dose per gram at 24 h but gradually decreased to below 1% by 7 days. Although a favourable tumour to blood ratio was seen as early as 24 h after administration, there were still 0.36 units $ml^{-1}$ active enzyme in plasma at 72 h. When SB43-galactose was injected 19 h after the $^{125}$I-conjugate the percentage of injected dose of the latter per gram of tumour remained almost unaltered (Figure 4). However, there was a greater than 2-fold decrease in the concentration of conjugate in the blood at 24 h after injection.

Mice injected with SB43-galactose showed higher tumour to blood ratios at all time points studied (Figure 5) but there was a higher uptake of radiolabelled conjugate in the liver

**Figure 1** Effect of SB43 on enzyme activity of A5B7-F(ab')2-CPG2 as measured by the spectrophotometric assay. SB43 (●), SB10 (■), rabbit anti-mouse IgG (▲).

**Figure 3** Effect of SB43 on plasma clearance of enzyme activity in nude mice bearing LS174T xenografts after admininstration (i.v.) of A5B7-F(ab')2-CPG2 (90 units of enzyme per mouse). **a**, Mice received conjugate followed 24 h later by saline (0.2 ml i.v.). **b**, Mice received conjugate followed 24 h later by 200 µg of SB43 (0.2 ml i.v.).

**Figure 4** Effect of SB43 galactose on clearance and localisation of $^{125}$I-A5B7-F(ab')2-CPG2 in mice bearing LS174T xenografts. Mice received conjugate (25 units of enzyme per mouse) and tumour (Δ) and blood (O) taken at 24, 48, 72 and 168 h after the conjugate injection. In another group of mice SB43 galactose (200 μg per mouse) was injected 19 h after the conjugate and tumour (▲) and blood (●) collected as before.

**Figure 5** Tumour: organ ratios obtained with $^{125}$I-A5B7-F(ab')2-CPG2 and the effect of SB43 galactose. No SB43-galactose (□), with SB43-galactose (▨).

and therefore a lower tumour to liver ratio during the first 24 h (Figure 5a, b, c). Tumour to lung ratios were higher at all time points studied.

### Discussion

A5B7-F(ab')2-CPG2 labelled with iodine-125 localises in LS174T xenografts within 24 h of injection. The percentage of the administered dose of conjugate in the tumour at 24 h was found to be dose dependent varying from about 4% ID $g^{-1}$ when 20 μg was injected to about 2% with higher doses of 150 μg (unpublished data). Although these levels are approximately half those obtained using unconjugated F(ab')2 fragment and five times less than those achieved with intact A5B7, it was nevertheless possible to achieve higher absolute levels of localised enzyme, compatible with current therapy experiments, by increasing the dose of conjugate while maintaining adequate tumour to blood ratios with SB43 aided clearance. In preliminary therapy studies (paper in preparation) we have used doses of conjugate equivalent to 25 units of enzyme activity, which corresponds to 150 μg of F(ab')2. At this dosage the rate of clearance of conjugated enzyme activity from blood, which tends to be somewhat slower than that reported for unconjugated antibody fragment alone (Rogers *et al.*, 1986; Buchegger *et al.*, 1983), may result from a very low level of circulating CEA ($<5 \text{ng ml}^{-1}$; Philben *et al.*, 1986) in this model. Complexes formed in an antigen excess would tend to be cleared more rapidly from the circulation by the reticulo-endothelial system. The latter situation, however, may be typical of many potential clinical applications where patients have a high blood CEA level.

In the mouse model studied, enzyme activity of CPG2 conjugated to A5B7 F(ab')2 persisted in blood for several days at a level which would result in conversion of prodrug with toxic effects. A safe level of enzyme in blood ($<0.3$ units $ml^{-1}$) is reached by 6 days when the level of conjugate in the tumour is about half of its value at 24 h. In order to make best use of the enzyme localised at the tumour site, clearance of enzyme from the circulation needs to be accelerated while keeping the tumour level unaltered.

In this study we have developed SB43, a monoclonal antibody which, in its native or galactosylated form, selectively inactivates CPG2 conjugates within minutes after injection. However, studies with $^{125}$I-labelled native SB43 have shown it to persist in the circulation at high levels for up to several days, resulting in a high non-specific uptake into LS174T xenografts. This could have the disadvantage of inactivating enzyme at the tumour site as well as in plasma. It is necessary, therefore, to restrict the inactivating antibody, SB43, from crossing the capillary endothelial membrane at the tumour site. We have achieved this by chemical modification of SB43 such that it clears from the circulation sufficiently rapidly to minimise non-specific uptake by tumour.

Since SB43 inactivates plasma CPG2 within minutes after injection, we have coupled it to cyanomethyl galactose (Mattes, 1987) which allows for immune complexes formed in antibody excess to be removed very rapidly from the circulation by the galactose-specific receptors in hepatic parenchymal cells (Thornburg *et al.*, 1980). By applying galactosylated SB43 we have been able to inactivate plasma CPG2 and accelerate clearance of $^{125}$I-labelled CPG2 conjugates from blood without appreciably affecting enzyme levels in the tumour. This has since been confirmed by measurements on localised active enzyme concentrations in excised tumours by *in vitro* turnover of prodrug (to be reported elsewhere). SB43 and its galactosylated derivative were also shown to be effective at accelerating plasma clearance and inactivating intact A5B7 conjugated carboxypeptidase G2.

The data presented here indicate that inactivation of the enzyme is very rapid and is probably more important than the clearance effect in reducing prodrug conversion in the blood. The mechanism of inactivation by SB43 may involve its binding at or near the active site of the enzyme or at a distant site causing a conformational change in the enzyme and loss of its biological activity.

The experiments described in this study are part of our continuing investigation into antibody directed enzyme prodrug therapy (ADEPT) in which: (1) antibody enzyme conjugate is allowed to achieve maximum localisation; (2) selective inactivation and clearance of enzyme from plasma is achieved by the galactosylated 'clearing' antibody; (3) prodrugs may be injected when enzyme conjugate at the tumour site is at its peak.

The three phase approach has several potential advantages in that by inactivating enzyme in blood, prodrugs can be safely administered while the tumour maintains a high concentration of active enzyme. Galactosylation of the 'clearing' antibody allows accelerated clearance from blood of complexed enzyme conjugate and, moreover, ensures that any uncomplexed SB43, which could otherwise inactivate enzyme localised at the tumour site, is also cleared. Use of the galactosylated 'clearing' antibody should enable the whole cycle of conjugate and prodrug to be repeated more frequently to achieve an enhanced therapeutic effect.

We are grateful to the Cancer Research Campaign for grant suport, to Drs R. Sherwood and R. Melton at Porton Down for a supply of carboxypeptidase G2 and to Joan Boden for skilled technical assistance.

### References

BAGSHAWE, K.D. (1987). Antibody directed enzymes revive anti-cancer prodrugs concept. *Br. J. Cancer*, **56**, 531.

BAGSHAWE, K.D., SPRINGER, C.J., SEARLE, F. & 4 others (1988). A cytotoxic agent can be generated selectively at cancer sites. *Br. J. Cancer*, **58**, 700.

BUCHEGGER, F., HASKELL, C.M., SCHREYER, M., SCAZZIGA, B., CARREL, S. & MACH, J.P. (1983). Radiolabelled fragments of monoclonal antibodies against CEA for localisation of human colon carcinoma grafted into nude mice. *J. Exp. Med.*, **158**, 413.

DUNCAN, R.J.S., WESTON, P.D. & WRIGGLESWORTH, R. (1983). A new reagent which may be used to introduce sulfhydrl groups into proteins, and its use in the preparation of conjugates for immunoassay. *Anal. Biochem.*, **132**, 68.

HUGHES, P., LOWE, C.R. & SHERWOOD, R.F. (1982). Metal ion-promoted binding of proteins to immobilized triazine dye affinity adsorbents. *Biochim. Biophys. Acta*, **700**, 90.

KOHLER, G. & MILSTEIN, C. (1975). Continuous cultures of fused cells secreting antibody of predefined specificity. *Nature*, **256**, 495.

MATTES, M.J. (1987). Biodistribution of antibodies after intraperitoneal or intravenous injection and effect of carbohydrate modifications. *J. Natl Cancer Inst.*, **79**, 855.

MCCULLOCH, J.L., CHABNER, B.A. & BERTINO, J.R. (1971). Purification and properties of carboxypeptidase G1. *J. Biol. Chem.*, **246**, 7207.

PHILBEN, V.J., JAKOWATZ, J.G., BEATTY, B.G. & 5 others (1986). The effect of tumour CEA content and tumour size on tissue uptake of indium 111-labelled anti-CEA monoclonal antibody. *Cancer*, **57**, 571.

ROGERS, G.T., HARWOOD, P.J., PEDLEY, R.B., BODEN, J. & BAGSHAWE, K.D. (1986). Dose dependent localisation and potential for therapy of F(ab')2 fragments against CEA studied in a human tumour xenograft model. *Br. J. Cancer*, **54**, 341.

SENTER, P.D., SAULNIER, M.G., SCHREIBER, G.J. & 4 others (1988). Anti-tumour effects of antibody-alkaline phosphate conjugates in combination with etoposide phosphate. *Proc. Natl. Acad. Sci. USA*, **85**, 4842.

SHERWOOD, R.F., MELTON, R.G., ALWAN, S.M. & HUGHES, P. (1985). Purification and properties of carboxypeptidase G2 from pseudomonas Sp strain RS16; use of a novel triazine dye affinity method. *Eur. J. Biochem.*, **148**, 447.

THORNBURG, R.W., DAY, J.F., BAYNES, J.W. & THORPE, S.R. (1980). Carbohydrate-mediated clearance of immune complexes from circulation. A role for galactose residues in the hepatic uptake of IgG-antigen complexes. *J. Biol. Chem.*, **255**, 6820.